# Lessons Learned from a Collaborative to Develop a Sustainable Simulation-Based Training Program in Neonatal Resuscitation: Simulating Success

**DOI:** 10.3390/children8010039

**Published:** 2021-01-12

**Authors:** Nandini Arul, Irfan Ahmad, Justin Hamilton, Rachelle Sey, Patricia Tillson, Shandee Hutson, Radhika Narang, Jennifer Norgaard, Henry C. Lee, Janine Bergin, Jenny Quinn, Louis P. Halamek, Nicole K. Yamada, Janene Fuerch, Ritu Chitkara

**Affiliations:** 1 CHOC Children’s Specialists Neonatology Division, Children’s Hospital Orange County, Orange, CA 92868, USA; narul@choc.org (N.A.); iahmad@choc.org (I.A.); Justin.Cain.Hamilton@CHOC.org (J.H.); 2 Neonatal Intensive Care Unit, Sharp Mary Birch Hospital for Women and Newborns, San Diego, CA 92123, USA; Rachelle.Sey@sharp.com (R.S.); Patricia.Tillson@sharp.com (P.T.); Shandee.Hutson@sharp.com (S.H.); 3 Valley Children’s Healthcare Division of Neonatology, Valley Children’s Hospital, Madera, CA 93636, USA; rnarang@valleychildrens.org (R.N.); jnorgaard@valleychildrens.org (J.N.); 4 Division of Neonatology, Department of Pediatrics, Stanford University School of Medicine, Stanford, CA 94305, USA; hclee@stanford.edu (H.C.L.); jmbergin@stanford.edu (J.B.); halamek@stanford.edu (L.P.H.); nkyamada@stanford.edu (N.K.Y.); jfuerch@stanford.edu (J.F.); 5 California Perinatal Quality Care Collaborative (CPQCC), Stanford, CA 94305, USA; jenny.quinn@neoqip.com; 6 NeoQIP (Neonatal Quality Improvement Performance) LLC, Martinez, CA 94553, USA; 7 Center for Advanced Pediatric and Perinatal Education (CAPE), Stanford, CA 94305, USA

**Keywords:** neonatal resuscitation, simulation, debriefing, quality improvement

## Abstract

Newborn resuscitation requires a multidisciplinary team effort to deliver safe, effective and efficient care. California Perinatal Quality Care Collaborative’s Simulating Success program was designed to help hospitals implement on-site simulation-based neonatal resuscitation training programs. Partnering with the Center for Advanced Pediatric and Perinatal Education at Stanford, Simulating Success engaged hospitals over a 15 month period, including three months of preparatory training and 12 months of implementation. The experience of the first cohort (Children’s Hospital of Orange County (CHOC), Sharp Mary Birch Hospital for Women and Newborns (SMB) and Valley Children’s Hospital (VCH)), with their site-specific needs and aims, showed that a multidisciplinary approach with a sound understanding of simulation methodology can lead to a dynamic simulation program. All sites increased staff participation. CHOC reduced latent safety threats measured during team exercises from 4.5 to two per simulation while improving debriefing skills. SMB achieved 100% staff participation by identifying unit-specific hurdles within in situ simulation. VCH improved staff confidence level in responding to neonatal codes and proved feasibility of expanding simulation across their hospital system. A multidisciplinary approach to quality improvement in neonatal resuscitation fosters engagement, enables focus on patient safety rather than individual performance, and leads to identification of system issues.

## 1. Introduction

Neonatal resuscitation is one of the most critical events in neonatal-perinatal medicine requiring a high level of individual skill and team performance. Resuscitation of a critically ill newborn cannot occur in a silo—it requires a team effort. Ineffective communication has been noted to play a role in almost 75% of cases of neonatal mortality or severe neonatal morbidity reported to the Joint Commission on Accreditation of Healthcare Organizations (JCAHO) [1,2]. Studies of real-life delivery room resuscitations have elucidated opportunities for improvement in behavioral skills, as well as lack of adherence to the recommended steps of the Neonatal Resuscitation Program (NRP) algorithm [3,4]. Simulation-based training for neonatal resuscitation in an immersive environment replicating a real clinical scenario provides an opportunity to improve behavioral and communication skills [5,6]. Notably, health care professionals (HCPs) who have completed simulation-based training in Adult Cardiopulmonary Life Support (ACLS) better adhere to resuscitation guidelines in the real-life clinical environment [7]. Thomas et al. translated principles of communication and teamwork behavior to neonatal resuscitation practice and developed a framework for assessing teamwork behavior using video recordings [8]. In a randomized trial testing the addition of teamwork training to NRP, those who received the teamwork training intervention exhibited more teamwork behavior than the control group [9,10]. NRP training has increasingly incorporated simulation-based training in these cognitive, technical, and behavioral skills with the aim of improving the quality of newborn resuscitation [11].

Neonatal resuscitation is complex and occurs infrequently. Team training aims to teach and support knowledge acquisition, and skills and attitudes that lead to optimal team performance. Simulation and debriefing methodology provide the tools to conduct team training with the primary goal of patient safety [2,12]. Simulations can be conducted in a simulation center or in situ (i.e., actual setting where participants work, for example Labor and Delivery). There are several benefits for health care teams to conduct simulations in situ [13]: adding realism; ability to identify systems errors or latent safety threats (LSTs) that could lead to changes in practice; and filling of gaps between knowledge and practice [14,15]. California Perinatal Quality Care Collaborative’s (CPQCC) Simulating Success program was designed to help participating hospitals implement an on-site, simulation-based neonatal resuscitation training program. In this report, we describe the experience of the first cohort of three hospitals to participate in the program. The structure of the remainder of this paper is such that the three institutions’ aims are each presented in the Methods along with the local context in which the project occurred, and the results of the project are then presented for each institution separately in the Results in corresponding order.

## 2. Materials and Methods

**Figure 1:**
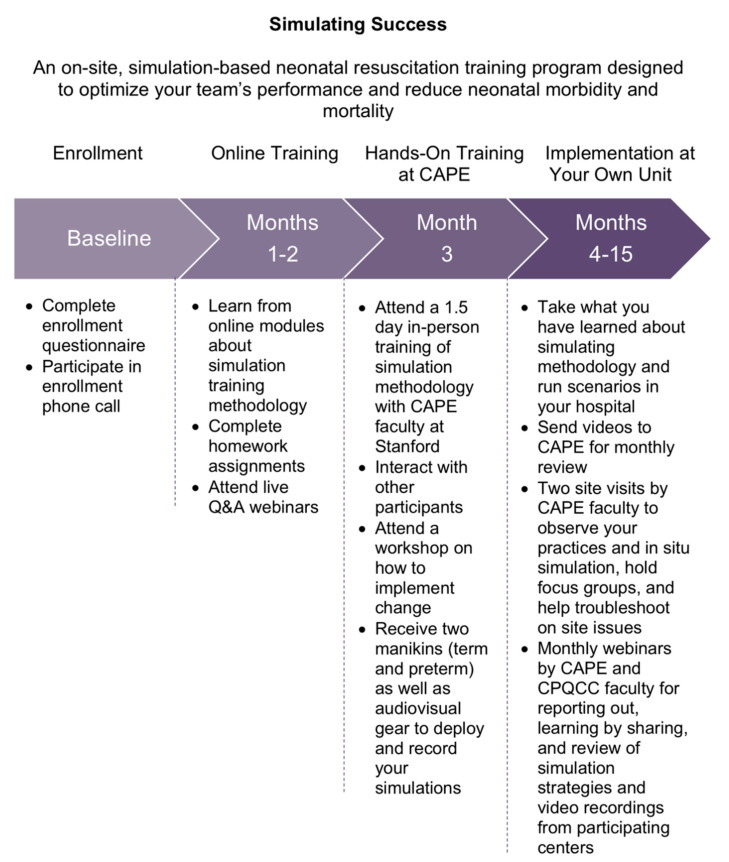
Timeline for CPQCC’s Simulating Success collaborative.

Simulating Success engaged hospitals over 15 month long periods that included three months of preparatory training followed by 12 months of implementation (Figure 1). Simulating Success was offered by CPQCC in partnership with the Center for Pediatric and Perinatal Education (CAPE) at Stanford University. Preparatory training consisted of an online didactic program followed by a 1.5 day, face-to-face training program at CAPE in the core principles of developing and conducting simulation-based training. The online didactic program was made available to an unlimited number of staff members at each site. Face-to-face training was attended by a maximum of three staff members from each site (referred to as the ‘multidisciplinary champion team’ for the remainder of this manuscript). Implementation at each site entailed ongoing in situ simulations followed by debriefings and monthly online check-ins with CAPE faculty, in addition to two site visits, for continued feedback and support.

The first cohort of three sites began in April 2018. Principles of quality improvement were incorporated throughout the collaborative with a focus on implementing Plan Do Study Act (PDSA) cycles. A quality improvement expert helped each site develop their Specific, Measurable, Applicable, Realistic, and Timely (SMART) Aim statements to target unit-specific needs. The resulting aim statements reflect the Simulating Success program goals of incorporating quality improvement tools and developing sustainable programs. Performance of the implementation teams as well as the clinical staff were used as potential measures of sustainability. This manuscript details the experience of these sites in implementing a simulation program at their respective hospitals (Children’s Hospital of Orange County (CHOC), Sharp Mary Birch Hospital for Women and Newborns (SMB) and Valley Children’s Hospital (VCH)).

### 2.1. CHOC

The CHOC Neonatal Intensive Care Unit (NICU) is a level IV, 104-bed NICU with a Surgical NICU, Small Baby Unit, Neuro NICU and Cardiac NICU within a free-standing Children’s hospital that also has a Pediatric Residency and Neonatal Fellowship program. Participation in Simulating Success was intended to enhance positive team behaviors during neonatal resuscitation that impact patient outcomes. The SMART aims were to (1) increase staff participation in at least one multidisciplinary simulation team training in the NICU from baseline of 0% to a target of 75% by June 2019; and (2) to improve patient safety through simulation exercises as demonstrated by a decrease in LSTs identified from a baseline of 4.5 per simulation to one over a period of six months, from January to June 2019.

LSTs are improvement goals identified during simulation exercises that have an impact on delivery of optimal care to the patient [14,16,17]. In situ simulations can help identify these knowledge gaps and reinforce positive team behaviors [18]. Knowledge gaps were further categorized as cognitive, technical, or behavioral. The CHOC instructor team used these debriefings to help identify LSTs. The CAPE Real-Time Debriefing Evaluation (DART) tool was used to assess the effectiveness of the debriefer(s) and participation of the learners during debriefing (Appendix A). Using the DART tool, a goal ratio of trainee responses to instructor questions plus statements is ideally >3:1. Participants were also asked to submit post-simulation surveys which included 13 questions related to understanding scenario learning objectives, duration and realism of the simulation, facilitators’ ability to encourage participation and knowledge base, participants’ confidence and psychological safety (Appendix B). The purpose of these were twofold: (1) to ideally increase the value of the program for trainees; and (2) to generate continuous data collection and share it with the Patient Safety Committee and hospital administration to engender support. The data was collected electronically via a QR code and entered into Research Electronic Data Capture (REDCap)—an existing data collection platform used by the hospital.

### 2.2. SMB

SMB is a hospital delivering nearly 8000 newborns each year in San Diego. The SMB NICU is a level III 84-bed unit with specialty care including a small baby program, neuro-intensive care program, and an advanced life support team that attends high-risk deliveries. The SMB NICU chose to participate in Simulating Success to address team competencies related to changing patient conditions and to improve communication skills in managing neonatal resuscitations. The team’s primary SMART aim was to implement and conduct monthly simulation and debriefing exercises in the NICU at SMB while increasing multidisciplinary participation by the end of the 18 month collaborative. Nursing and respiratory departments added a requirement for each person to participate in at least two simulation and debriefing events per year for their annual performance evaluation. Historically, “mock codes” in this NICU did not focus on behavioral competencies required for improving teamwork, collaboration, and communication. In addition, the team wanted to improve staff confidence in managing changing patient conditions including appropriate use of positive pressure ventilation to avoid an unnecessary full resuscitation leading to intubation and/or chest compressions.

### 2.3. VCH

The Neonatal Service Line of Valley Children’s Healthcare includes an 88-bed level IV Regional NICU located at the free-standing children’s hospital in Madera, a 14-bed level III community NICU in Fresno, an 8-bed level II community NICU in Merced, and a second 6-bed level II NICU in Hanford. Given the complexity of multiple locations, staff composition and size, and critical nature of the patient population, VCH chose to participate in Simulating Success with the goal of improving team clinical and communication skills through simulation-based training. The team’s goal was to increase the number of simulations offered and to improve the comfort level and skill set of staff performing resuscitation for infants in all units through effective simulation and debriefing. The first SMART aim was to run two simulation events per month at Regional NICU, two simulation events per month at one of the level II NICUs, and one quarterly simulation event at the other level II NICU by October 2020. The second SMART aim was to have each member of the core simulation team perform two debriefs per quarter and participate in two of the monthly simulation events.

## 3. Results

The following is a report of results by each site to date. Each hospital is discussed separately since each had different SMART aims. It is important to note that this analysis does not include analyses of patient outcomes, which continues to be tracked at the time of this writing.

### 3.1. CHOC

The multidisciplinary champion team at CHOC was comprised of a neonatologist, nurse educator and respiratory therapist. A total of 10 HCPs (four physicians, three nurses, three respiratory therapists) completed the online video training. Video-recorded simulations and debriefings were started in July 2018. The first 10 recordings served as a baseline to inform the creation of the first simulation improvement bundle which included (1) use of standardized briefings prior to simulation; (2) NRP education classes and skills workshops; (3) consistent use of the debriefer rating tool from CAPE; (4) use of debriefing the debriefer; and (5) addition of a simulation specialist to help conduct these team exercises. This bundle was implemented in January 2019 with modifications during multiple PDSA cycles. A total of 38 simulation exercises were completed in situ on Labor and Delivery and the NICU or in a simulation lab.

#### 3.1.1. SMART Aim #1

Increase staff participation. A total of 73% of physicians, 48% of nurses, and 100% of respiratory therapists were exposed to at least one simulation exercise through June 2019.

#### 3.1.2. SMART Aim #2

**Figure 2:**
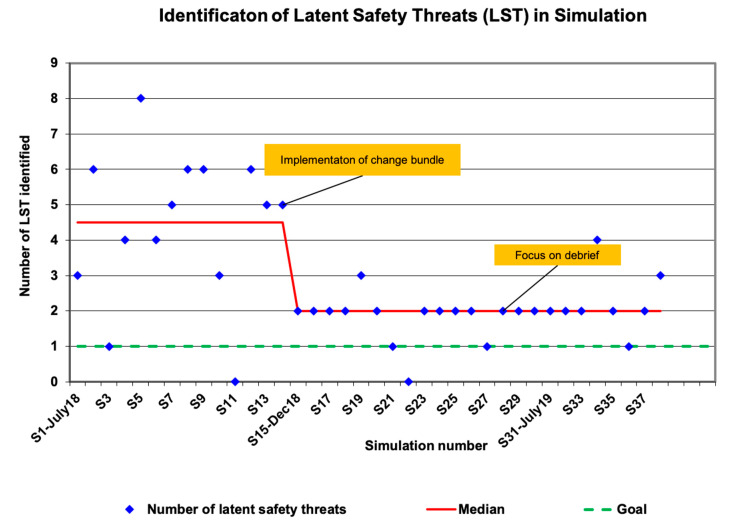
LSTs identified during CHOC simulations over time. Of note, there was a significant shift in the median (>8 consecutive data points below the median) during PDSA cycle 2 towards the goal of ≤1.

Decrease LSTs. After implementation of the simulation improvement bundle, LSTs decreased and there was a shift in the median to two LSTs per simulation (Figure 2). Of the LSTs identified, 57% were found to involve technical (e.g., lack of knowledge on usage of laryngeal mask) and 31%, behavioral issues (e.g., lack of role assignment) while 6.4% were attributable to cognitive issues (e.g., knowledge about delayed cord clamping) and 5.4% to system errors (e.g., failed pages).

#### 3.1.3. Other Notable Results

**Figure 3:**
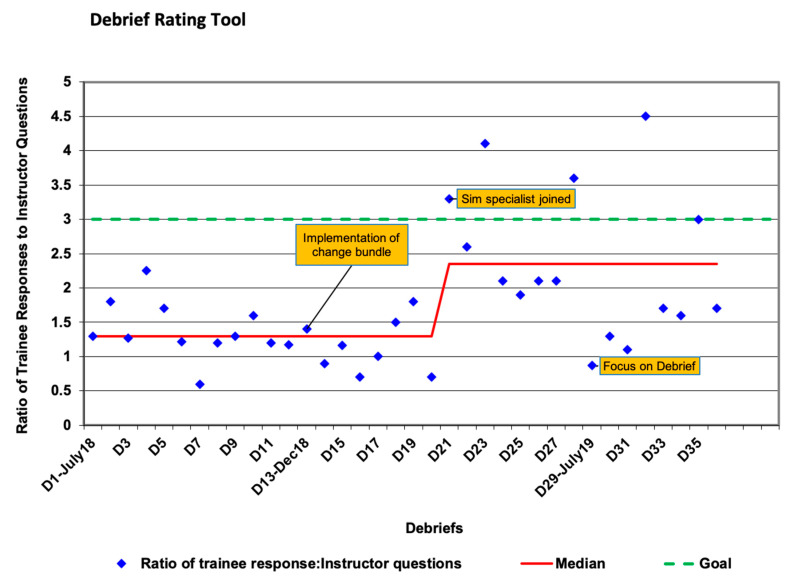
CAPE Real-Time Debriefing Evaluation (DART) scores for CHOC simulations over time. Of note, median shows improvement over time with a shift in median towards a goal ratio of >3:1.

DART scoring revealed improvement over time towards the goal ratio of 3:1 [trainee responses: instructor questions + statements] (Figure 3). Of the 61 post-simulation surveys sent, 56 were completed; 90% of participants strongly agreed or agreed with the objectives of the program. These objectives were to provide a realistic simulated multidisciplinary team training experience in a constructive and psychologically safe learning environment and with ongoing feedback for improvement from participants. Notably, 89% of the participants believed the debriefing was constructive, 92% felt safe participating in the debrief and 90% wanted to experience more simulation sessions. In response to early qualitative feedback on sessions sometimes being overly long, subsequent sessions were adjusted by having a set time for debriefing.

#### 3.1.4. Lessons Learned

The collaborative process of CPQCC’s Simulating Success Program provided many benefits including the opportunity to: (1) learn from national experts; (2) share challenges and successes; (3) learn from and adapt to different settings; (4) share tools such as confidentiality agreement, surveys and clinical scenarios amongst the sites. This enabled participating sites to appreciate the power of learning from one another. The collaborative approach also helped the team to develop an urgency for change at the institutional level, encouraged friendly competition and fostered accountability. Monthly review of video recordings of the simulation and debriefings with our mentors at CAPE gave the team several opportunities to improve. Systems issues identified during these exercises led to process changes in how codes were called overhead in the NICU and replacing pagers to phones for Labor and Delivery to eliminate missed calls from failed pages.

#### 3.1.5. Challenges Faced

Common difficulties in implementing CHOC’s simulation program included time allocation, turnover of trained staff, and achieving the desired number of simulation sessions per month. There were several competing projects in the unit that made it difficult for the facilitators to allocate time for the goal number of sessions per month. This also made the growth of the team difficult to achieve. High census during October through December 2019 limited the team’s ability to continue with a goal number of simulation sessions. Monthly webinars and face to face meetings helped CHOC’s team trouble shoot some of these hurdles.

### 3.2. SMB

**Figure 4:**
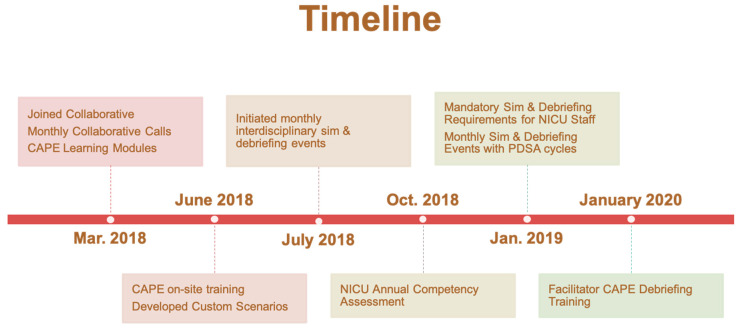
SMB Simulating Success project timeline.

The multidisciplinary champion team at SMB was comprised of a neonatologist, clinical nurse specialist, a NICU supervisor and respiratory therapist. A total of 15 HCPs completed the online video training. The team developed a variety of custom NICU scenarios based on actual code events that occurred in Labor and Delivery and in the NICU. Beginning in July 2018, the team began video recording of simulations and debriefings. A timeline of the progression of the project is depicted in Figure 4. Simulation and debriefing events were held monthly for both day shift and night shift. Neonatologists and neonatal nurse practitioners were encouraged to participate in at least one simulation. Initial survey results demonstrated overwhelmingly positive results from HCPs who participated in these simulation and debriefing events. Forty-two HCPs responded to the initial survey; nurses and respiratory therapists represented 85% of the respondents. These respondents had moderate level of experience in their specialty (14.0 years + 11.7). Results demonstrated that participants had increased confidence in communicating during an emergency, increased ability to function as an essential team member during a code, and increased ability to voice a concern during a critical situation (Appendix C).

Early in the implementation phase, the core team realized the need for additional simulation/debriefing facilitators and champions. The core team used a “train-the-trainer” approach to onboard additional facilitators. The train-the-trainer approach was not sufficient—additional preparation specific to effective debriefing was necessary. NICU nurses and respiratory therapists, mostly NRP instructors and advanced life support nurses who attend high-risk deliveries, volunteered to participate as champions. They each completed the CAPE online training course, “Strategies for Debriefing Health Care Scenarios”.

#### 3.2.1. SMART Aim #1

SMART Aim #1: Increase participation. A total of 100% of nurses (*n* = 205) and respiratory therapists (*n* = 40) participated in at least two simulation events within the first year. Participation in a minimum number of simulation and debriefing events were added to the annual competency requirements for both nurses and respiratory therapists which enabled achievement of this SMART aim. Throughout the 18 month participation in the collaborative, SMB utilized the PDSA process for the quality improvement framework.

#### 3.2.2. Lessons Learned

The use of video to enhance CAPE’s debriefing techniques allowed everyone to speak up and recognize areas for improvement. The debriefing technique which was new to SMB involved a flipped approach where the debriefers ask prompting questions or statements to elicit discussion from the participants thereby allowing the participants to identify strengths and opportunities for themselves. As a team, they critique their own performance and identify behaviors that they should reinforce and opportunities for improvement. Building effective, realistic scenarios using real-life events that have occurred at SMB helped staff identify and intervene with changing patient conditions. Strategies were identified to increase interdisciplinary team participation including adaptations to various provider schedules.

Effective debriefing has been key for successful learning. Some focus areas identified were the need for increased communication, consistently designating a team leader, knowledge of when to call for help and closed loop communication. Less experienced staff, especially on night shift, have expressed an increased confidence in ability to respond during an emergency. They have also increased their ability to recognize patient deterioration through simulation. Staff comments have been very positive. For example, staff comments include “This is the best mock code I have ever participated in; it is so realistic”; “This is such a great learning environment”; and “I really like how realistic this mock code is.” In addition to the monthly simulation and debriefing sessions, newly learned debriefing skills have been incorporated into the review and evaluation of video-recorded neonatal resuscitations.

For more than 15 years, the SMB NICU team has used video resuscitation review as an ongoing quality improvement project to improve delivery room resuscitation. Using the debriefing model to discuss areas for improvement identified by the team has been highly effective. This process has proven beneficial in identification of a team leader, effective communication, and delegation of tasks. It has created an atmosphere that focuses more on team processes as opposed to individual performance. Given the technical components of simulation and debriefing (running video equipment, high-fidelity mannequin, and time commitment for set up and tear down), the goal was to have at least two facilitators at each debriefing. After initial experience, three facilitators were found to be ideal: one to direct the scenario and debriefing, one to control the high fidelity simulator, and one that manages the technical aspects of the debriefing with video equipment. In addition, the third person acted as a confederate as needed or as an additional debriefer.

#### 3.2.3. Challenges Faced

Identified challenges with in situ simulation included (1) lack of dedicated space to conduct simulations and debriefings and inadequate space to store all the equipment; (2) technical and time consuming challenges of setting up for each simulation and debriefing; (3) interruption of simulations/debriefings by events occurring in the unit (i.e., high census, high acuity); (4) ensuring adequate participation. Monthly multidisciplinary simulation and debriefing sessions were offered on each shift. Initially, they were offered on weekends as well, but there was less participation on weekends. Varying days and times simulation/debriefing sessions were offered throughout the month encouraged staff participation. Dates and times of all scheduled simulation and debriefing sessions were emailed and posted in advance allowing staff to plan ahead. Early in the process, it was difficult to achieve physician and nurse practitioner participation since scheduled times mostly occurred in the afternoon or evenings when HCPs had already completed their shift for the day. In order to increase physician and nurse practitioner participation, session times were moved to accommodate rounding schedules and ensure multiple physicians were present on the unit to attend simulations and provide patient care as needed. They were also timed around typical breaks for nurses and respiratory therapists to enable them to participate.

### 3.3. VCH

The multidisciplinary champion team at VCH was comprised of two clinical nurse specialists and a neonatologist.

#### 3.3.1. SMART Aim #1

**Figure 5:**
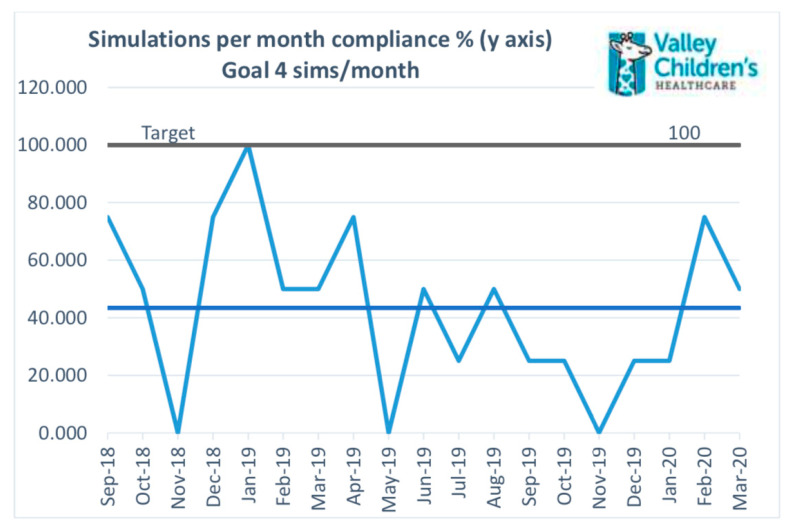
Average compliance with the goal of four simulations at VCH.

Increasing number of simulations. The global aim of increasing the number of simulations conducted in the Neonatal Service Line of Valley Children’s Healthcare was achieved although the monthly and quarterly targets for the number of simulations per month were not always met. The team has been able to significantly improve the quantity and quality of simulation training. The average compliance with the aim of four simulations per month was 40% (Figure 5).

#### 3.3.2. SMART Aim #2

Performing debriefings. Due to core team turnover, only the five consistent team members for compliance with number of simulations conducted per month and the number of debriefs per quarter have been tracked. The compliance of team members conducting two simulations per month ranges from 0% to 80% with a median of 40%. The compliance of team members conducting two debriefs per quarter ranges from 0% to 80% with a median of 60%. Areas of improvement have been identified and the team is continuing with data gathering and analysis. A follow-up staff survey in fall of 2020 will be conducted.

#### 3.3.3. Other

As a part of the first PDSA cycle, the team identified a need to elicit baseline satisfaction and self-assessment of skill and confidence in code situations; therefore, the team developed a survey to be completed by all clinicians in the NICU (physicians, neonatal nurse practitioners, nurses and respiratory therapists). Based on the survey results, the team developed the following aims (Appendix D):

- Increase participation in 2–3 simulations/year from 18% to 40%;
- Decrease dissatisfaction with simulation experience to <10%;
- Decrease lack of confidence in the ability to participate in a neonatal code blue to <10%;
- Decrease lack of confidence in the ability to lead a neonatal code blue to <10%;
- Decrease lack of comfort with communication skills required during a neonatal resuscitation to <5%.

#### 3.3.4. Lessons Learned

By careful scenario design and planning, a major component of Simulating Success, the VCH team has been able to capitalize on two other major quality improvement initiatives—reduction in unplanned extubations and implementation of Golden Hour Guidelines for premature infants. Simulation expanded into several other arenas of staff development and training, including competency assessment in resuscitation, transport and clinical skills. CAPE simulation techniques and scenarios have been used to deliver resuscitation training at multiple referring facilities as well. The debriefing model taught by CAPE and use of video for debriefing was new to the VCH team. Over the course of the collaborative, team members have grown from being unsure to developing real confidence in their ability to effectively debrief. A robust multidisciplinary team (clinical nurse specialist, nurse, respiratory therapists, neonatologist, and neonatal outreach coordinator) has enabled them to fine tune the different aspects of the simulation scenarios. Simulation training aligns with the safety goals of VCH which has facilitated leadership support in both equipment purchases, staff time, and designated space.

#### 3.3.5. Challenges Faced

First, high census/acuity impacts the team’s ability to conduct scheduled simulations thereby making it difficult to ensure team availability and staff participation. Second, the logistics of conducting simulations at four locations (Regional NICU and three satellite NICUs) with one simulation staff remains a challenge. Third, the team experienced turnover in several key roles requiring the onboarding and training of new simulation team members which was time consuming. Finally, constantly moving equipment due to a lack of dedicated space to conduct simulations/debriefings contributed to the inability to fully achieve the team’s goals.

## 4. Discussion

As with any team event, the more that team members practice together, the better they perform together [19]. When the core principles for simulation and debriefing are followed, it is possible to deliver safe, effective and efficient patient care [20]. Simulation improves neonatal resuscitation and patient safety [21]. Participation in CPQCC’s Simulating Success has shown that a multidisciplinary approach to quality improvement creates more engagement, enables focus to be directed towards patient safety rather than individual performance and leads to identification of system issues. To highlight one example, in the CHOC experience, a major systems issue identified through simulations was the paging method by which codes were called in the NICU. This led to inconsistencies in staff response. This issue was referred to leadership in CHOC’s Code White Committee which recommended the change to overhead code white calls, consistent with hospital wide code white calls, for appropriate staff response. Similarly, missed pages during simulation sessions were documented in Labor and Delivery which led to a change to iPhones for teams to respond and communicate in real-time.

### 4.1. Future Directions: CHOC

In 2020, with ongoing simulation exercises, our aim is to improve (1) long-term outcomes including decreasing the rate of chronic lung disease (CLD) from 25% to 20% and severe intraventricular hemorrhage (IVH) from 15.9% to 12%; and (2) short-term measures of decreasing the frequency of Apgar score <8 at five minutes, decreasing the number of intubation attempts in the delivery room, decreasing the frequency of cardiopulmonary resuscitation in the delivery room, and decreasing the time to leave the delivery room to the NICU. Simulating Success has been an impetus to expanding the simulation program at CHOC to involve other specialties including emergency medicine, pediatric intensive care, cardiovascular intensive care and hospital medicine. A temporary simulation center with basic infrastructure for a simulation room and a debriefing room with audiovisual capacities was built to conduct simulation lab exercises. The program at CHOC is able to use the space for procedural skill training for incoming attendings, residents, and fellows. The team at CHOC continues to collect data on resource utilization to emphasize the demand for this program to hospital leadership. A core simulation team has been established which meets monthly. A website for the simulation program has been created that can be accessed through the hospital intranet that contains the mission, training modules, confidentiality agreement, liability and request forms, and a list of facilitators and mentors. A three-tier facilitator program is being developed to help facilitators advance their skills in order to become mentors and grow the program. A tiered approach was created so as to ensure the quality of the program. Several facilitators have completed the online debriefing program through CAPE. Currently, neonatal and pediatric critical care are the main specialties involved in simulation with a plan to develop a simulation task force for both of these divisions. There will be ongoing education through reviews and webinars for facilitators on simulation methodology similar to the CAPE online debriefing course in order to further hone skills. A common debriefing language will be used for all simulations performed in the organization to integrate it as part of our culture of care.

### 4.2. Future Directions: SMB

In 2020, the SMB core team of facilitators will meet quarterly to review videos of their debriefings with the intent to improve facilitating effective simulation scenarios and debriefing using a “debriefing the debriefer” model. The process used by CAPE and CPQCC of providing insightful feedback during face to face sessions has helped to train the team’s facilitators. A train-the-trainer model has been implemented whereby experienced debriefers are scheduled with newly trained debriefers. Finally, in order to continue to enhance the program and assess its effectiveness among each of the disciplines, a pre–post-survey to elicit ongoing feedback has been created.

### 4.3. Future Directions: VCH

The team at VCH is working on budget planning to schedule simulations six months in advance, assess for feasibility to schedule staff and conduct multiple simulation events in a day. They aim for shared accountability amongst all simulation team members for set up, planning and implementation. They hope to train additional team members to replace those that have moved into other roles and continue the professional development of existing members. The team is eager to continue its work with their partners in Simulating Success through the sharing of experiences, scenarios and best practices.

### 4.4. Limitations

Our paper is limited in that these are the experiences of three institutions, presented as case studies, with differing aims and outcome measures. This limits the conclusions that can be drawn regarding the value and impact of the program; however, we hope that the narrative descriptions of these experiences can be helpful to institutions working on simulation and debriefing implementation for neonatal resuscitation.

## 5. Conclusions

In situ simulation aids in identifying system issues and focuses on improving patient safety [22]. Our paper describes the experiences of three sites with some similar, but also different contexts and results of implementing in situ simulation programs for neonatal resuscitation. Lessons learned will inform each institution’s continued progress, but may also be useful for others embarking on similar implementation projects. A goal of the implementation for all three institutions was to potentially affect systems change, and ultimately lead to improved patient care and outcomes. Each hospital in this collaborative had their own set of challenges and approached their simulation training and implementation methods based on the unique needs and goals of their unit. However, there were commonalities in barriers, such as adapting to fluctuations in clinical acuity and census and having consistent, dedicated team members with time allotted for the program. Establishing a strong foundation in simulation methodology, developing debriefing skills, ensuring multidisciplinary champions for implementation, and following a quality improvement framework are key components in order to achieve the goals of enhancing teamwork in neonatal resuscitation in order to improve clinical outcomes.

## Figures and Tables

**Figure A1:**
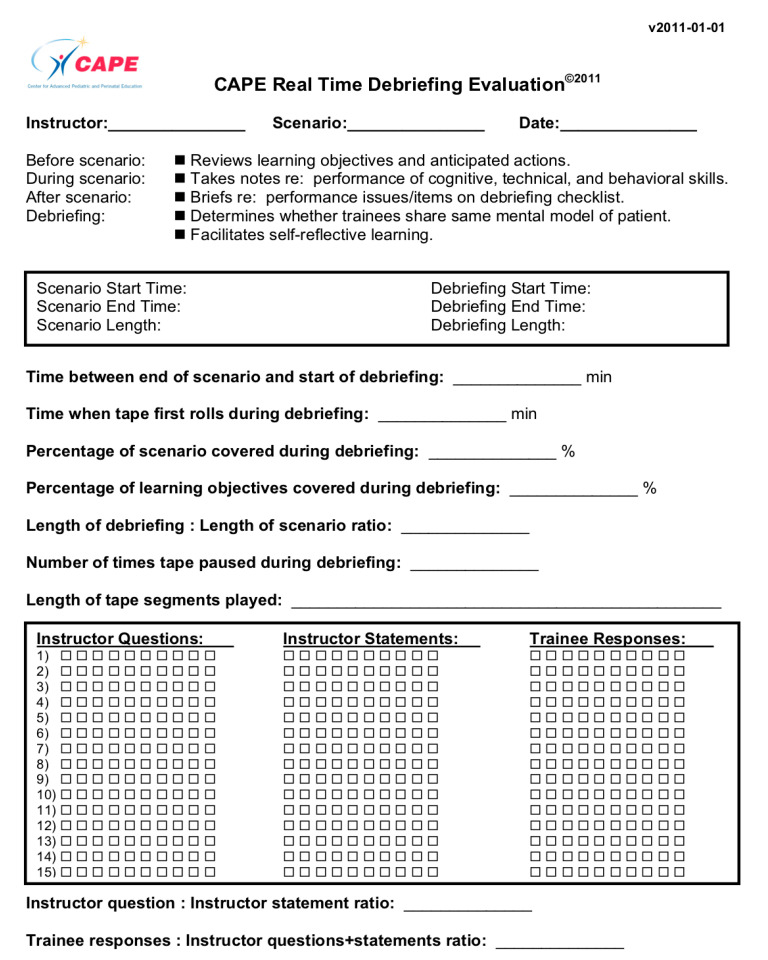
DART tool.

## Data Availability

The data presented in this study may be available on request from the corresponding author. The data are not publicly available due to privacy concerns and some data not consented to be shared.

## Appendix B

**Table A1:** CHOC post-simulation evaluation and survey results.

**1a. I clearly understood the purpose and objectives of this simulation exercise:**		
**Answer Choices**	**Percentage %**	**Responses**
Strongly Agree	59	33
Agree	32	18
Disagree	2	1
Strongly Disagree	7	4
Total		56
**1b. The scenario represented a real-life situation:**		
**Answer Choices**	**Percentage %**	**Responses**
Strongly Agree	59	33
Agree	34	19
Disagree	0	0
Strongly Disagree	7	4
Total		56
**1c. The debriefing discussion after the simulation was constructive:**		
**Answer Choices**	**Percentage %**	**Responses**
Strongly Agree	68	38
Agree	21	12
Disagree	4	2
Strongly Disagree	7	4
Total		56
**1d. I felt that the learning environment was safe:**		
**Answer Choices**	**Percentage %**	**Responses**
Strongly Agree	71	40
Agree	21	12
Disagree	0	0
Strongly Disagree	7	4
Total		56
**1e. I would like to participate in another simulation experience:**		
**Answer Choices**	**Percentage %**	**Responses**
Strongly Agree	52	29
Agree	38	21
Disagree	2	1
Strongly Disagree	7	4
Total		55
**2a. As a result of the simulation experience, I have increased my ability to anticipate the needs of other team members:**		
**Answer Choices**	**Percentage %**	**Responses**
Strongly Agree	52	29
Agree	39	22
Disagree	2	1
Strongly Disagree	7	4
Total		56
**2b. As a result of the simulation experience, I have increased my ability to communicate efficiently with other team members:**		
**Answer Choices**	**Percentage %**	**Responses**
Strongly Agree	50	28
Agree	41	23
Disagree	2	1
Strongly Disagree	7	4
Total		56
**3a. The facilitators were knowledgeable about patient care in the scenario:**		
**Answer Choices**	**Percentage %**	**Responses**
Strongly Agree	64	36
Agree	29	16
Disagree	0	0
Strongly Disagree	7	4
Total		56
**3b. The facilitators were well prepared for the session:**		
**Answer Choices**	**Percentage %**	**Responses**
Strongly Agree	68	38
Agree	21	12
Disagree	4	2
Strongly Disagree	7	4
Total		56
**3c. The facilitators encouraged active participation during the debriefing session:**		
**Answer Choices**	**Percentage %**	**Responses**
Strongly Agree	70	39
Agree	23	13
Disagree	0	0
Strongly Disagree	7	4
Total		56
**4a. The scenario was presented in a realistic environment:**		
**Answer Choices**	**Percentage %**	**Responses**
Strongly Agree	59	33
Agree	36	20
Disagree	0	0
Strongly Disagree	5	3
Total		56
**4b. The location and time worked for me:**		
**Answer Choices**	**Percentage %**	**Responses**
Strongly Agree	46	26
Agree	36	20
Disagree	13	7
Strongly Disagree	5	3
Total		56
**4c. The session lasted about the right time:**		
**Answer Choices**	**Percentage %**	**Responses**
Strongly Agree	54	30
Agree	36	20
Disagree	5	3
Strongly Disagree	5	3
Total		56
**5. What were the positive aspects of the experience?**		
**6. What changes would you recommend to improve future simulation experiences?**		
**7. Additional Comments: Examples below:** “Created a great learning environment and the multidisciplinary aspect was helpful and made it more realistic.” “I liked the discussion of the medical aspect and the crisis resource management for each simulation.” “Non-judgmental, Informative.” “The situation was more real than other simulations I have been a part of in the past 3 years.” “Being able to work through common hiccups with critical situations, work through the and debrief afterwards improved confidence in being at bedside.”		

## Appendix C

**Table A2:** SMB post-simulation evaluation and survey results.

**1. What is your current role in the NICU:**			
**Answer Choices**	**Percentage %**	**Responses**	
Advanced Life Support Nurse	3.6	2	
Registered Nurse	67.3	37	
Respiratory Therapist	20.0	11	
Physician	3.6	2	
Nurse Practitioner	1.8	1	
Other	3.6	2	
Total		55	
		**Mean**	**SD**
**2. # Years in Current Role**		14.02	10.6
**3. # Years in Current Role at SMB**		9.82	8.5
**4. # Sim & Debrief sessions in past year**		2.33	1.9
**5. # Code Pink Events in past year**		0.68	1.1
**6. Since participating in NICU Simulation and Debriefing, I have become more confident in my ability to communicate effectively during an emergency:**			
**Answer Choices**	**Percentage %**	**Responses**	
Strongly Agree	50	27	
Agree	50	27	
Disagree	0	0	
Strongly Disagree	0	0	
Total		54	
**7. Since participating in NICU Simulation and Debriefing, I am able to recognize and intervene in a rapidly changing patient situation:**			
**Answer Choices**	**Percentage %**	**Responses**	
Strongly Agree	46.3	25	
Agree	53.7	29	
Disagree	0	0	
Strongly Disagree	0	0	
Total		54	
**8. I am able to voice my concerns during a critical situation:**			
**Answer Choices**	**Percentage %**	**Responses**	
Strongly agree	63.0	34	
Agree	37.0	20	
Disagree	0	0	
Strongly disagree	0	0	
Total		54	
**9. Overall, I am confident in my ability to participate as an essential team member in a neonatal code:**			
**Answer Choices**	**Percentage %**	**Responses**	
Strongly agree	40.7	22	
Agree	59.3	32	
Disagree	0	0	
Strongly disagree	0	0	
Total		54	
**10. The debriefing process is effective in aiding the team and learner to reflect on team performance and identify potential learning opportunities:**			
**Answer Choices**	**Percentage %**	**Responses**	
Strongly agree	70.4	38	
Agree	29.6	16	
Disagree	0	0	
Strongly disagree	0	0	
Total		54	

## Appendix D

**Table A3:** VCH post-simulation evaluation and survey results.

**1. Number of simulations (excluding NRP) you have participated in during the last year:**		
**Answer Choices**	**Percentage %**	**Responses**
0–1	79	110
2–3	18	24
4+	3	4
Total		139
**2. I am satisfied with simulation experience:**		
**Answer Choices**	**Percentage %**	**Responses**
Strongly disagree	7	9
Disagree	14	19
Agree	61	83
Strongly agree	19	26
Total		137
**3. I am confident in my ability to participate in a neonatal code blue:**		
**Answer Choices**	**Percentage %**	**Responses**
Strongly disagree	4	5
Disagree	11	15
Agree	56	77
Strongly agree	30	41
Total		138
**4. I am confident in my ability to be a team leader during a neonatal code blue:**		
**Answer Choices**	**Percentage %**	**Responses**
Strongly disagree	8	11
Disagree	28	38
Agree	38	53
Strongly agree	26	36
Total		138
**5. I am confident with my ability to perform skills of neonatal resuscitation (i.e., positive pressure ventilation, chest compression, dosing and administering medications):**		
**Answer Choices**	**Percentage %**	**Responses**
Strongly disagree	1	2
Disagree	7	9
Agree	59	82
Strongly agree	33	46
Total		139
**6. I am confident in my ability to anticipate clinical changes and interventions needed at bedside:**		
**Answer Choices**	**Percentage %**	**Responses**
Strongly disagree	1	2
Disagree	2	3
Agree	57	79
Strongly agree	40	55
Total		139
**7. I am comfortable with my communication skills required during a neonatal resuscitation:**		
**Answer Choices**	**Percentage %**	**Responses**
Strongly disagree	2	3
Disagree	8	11
Agree	58	80
Strongly agree	32	45
Total		139
